# Determination of Pork Meat Storage Time Using Near-Infrared Spectroscopy Combined with Fuzzy Clustering Algorithms

**DOI:** 10.3390/foods11142101

**Published:** 2022-07-14

**Authors:** Qiulin Li, Xiaohong Wu, Jun Zheng, Bin Wu, Hao Jian, Changzhi Sun, Yibiao Tang

**Affiliations:** 1 Institute of Talented Engineering Students, Jiangsu University, Zhenjiang 212013, China; qiulin_lee@163.com (Q.L.); scz_0307@163.com (C.S.); yibiaot@163.com (Y.T.); 2 School of Electrical and Information Engineering, Jiangsu University, Zhenjiang 212013, China; wxh419@ujs.edu.cn; 3 High-Tech Key Laboratory of Agricultural Equipment and Intelligence of Jiangsu Province, Jiangsu University, Zhenjiang 212013, China; 4 Department of Electrical and Control Engineering, Research Institute of Zhejiang University-Taizhou, Taizhou 318000, China; 5 Department of Information Engineering, Chuzhou Polytechnic, Chuzhou 239000, China; wubin2003@163.com; 6 China Railway Construction Electrification Bureau Group Co., Ltd., Beijing 100020, China; jh122050403@163.com

**Keywords:** pork meat, near-infrared (NIR) spectroscopy, MSC, OLDA, fuzzy C-means clustering, K-harmonic means clustering, GK clustering

## Abstract

The identification of pork meat quality is a significant issue in food safety. In this paper, a novel strategy was proposed for identifying pork meat samples at different storage times via Fourier transform near-infrared (FT-NIR) spectroscopy and fuzzy clustering algorithms. Firstly, the FT-NIR spectra of pork meat samples were collected by an Antaris II spectrometer. Secondly, after spectra preprocessing with multiplicative scatter correction (MSC), the orthogonal linear discriminant analysis (OLDA) method was applied to reduce the dimensionality of the FT-NIR spectra to obtain the discriminant information. Finally, fuzzy C-means (FCM) clustering, K-harmonic means (KHM) clustering, and Gustafson–Kessel (GK) clustering were performed to establish the recognition model and classify the feature information. The highest clustering accuracies of FCM and KHM were both 93.18%, and GK achieved a clustering accuracy of 65.90%. KHM performed the best in the FT-NIR data of pork meat considering the clustering accuracy and computation. The overall experiment results demonstrated that the combination of FT-NIR spectroscopy and fuzzy clustering algorithms is an effective method for distinguishing pork meat storage times and has great application potential in quality evaluation of other kinds of meat.

## 1. Introduction

Pork meat is rich in essential amino acids, nitrogen, inorganic salt, niacin, and vitamins. It can improve the health of the human body, such as promoting the growth of teenagers and reducing the risk of disease (prevention of senile osteoporosis, iron deficiency anemia, diabetes, pneumonia, and heart cerebrovascular disease [1]). As an edible meat, the freshness of pork meat has attracted extensive attention. However, expired and spoiled pork meat affects not only the taste and nutritional needs but also human health. To gain a higher profit, lawless merchants have deeply processed low-quality materials to disguise and sell them, whose substandard pork meat contains a large number of carcinogens such as nitrosamines and polycyclic aromatic hydrocarbons. Additionally, there are a wide variety of reasons for pork spoilage, for instance, storage temperature, humidity, air, pH value [2,3,4,5], and so on. In addition, the pork meat storage time is also a vital factor leading to pork spoilage. Pork meat is vulnerable to enzymes and microorganisms during storage, transportation, and processing, which decomposes and produces toxic chemical components, including histamine, tyramine [6], and putrescine. These decomposition activities intensify as the pork meat is stored for longer periods of time, eventually leading to spoilage and inedibility. Therefore, quick and accurate identification of the meat storage time can provide a significant basis for the determination of pork meat freshness.

The determination of pork meat freshness has always been a significant issue in food safety. Traditional meat freshness detection mainly includes sensory evaluation and physical and chemical detection. Sensory evaluation is easily influenced by subjective factors. In addition, physical and chemical detection is used to determine the content of pork components such as TVB-N, pH, fat, sugar, and protein. In order to quickly and accurately detect the freshness of pork meat, Qu et al. utilized near-infrared (NIR) spectroscopy combined with multi-index statistical information fusion (MSIF) to quantitatively detect the TVB-N content and pH value [7]. Huang et al. used Fourier transform near-infrared (FT-NIR) spectroscopy in the range of 4000–10,000 cm^−1^ with synergy interval partial least square (SI-PLS) and back-propagation artificial neural network (BP-ANN) to determine the contents of TVB-N, total fat, total sugar, and protein in pork meat [8]. Zhuang et al. used fluorescence hyperspectral imaging (HSI) and partial least squares regression (PLSR) to model the analysis of TVB-N, pH, L *, a *, and b * of frozen meat [9]. Chen et al. used the successive projections algorithm (SPA), gray level co-occurrence matrix (GLCM), and PLSR to enhance the hyperspectral imaging prediction ability of the K value in pork meat [10]. Kucha et al. attempted to use a miniaturized and portable near-infrared (NIR) spectroscopy and weighted regression coefficients-partial least square regression (RC-PLSR) to rapidly detect thiobarbituric acid reactive substances (TBARS) values in minced meat to assess the freshness [11].

The use of NIR spectroscopy to analyze samples is fast, non-destructive, efficient, and accurate, with no consumption of chemical reagents and no environmental pollution [12,13,14,15]. In the past, NIR spectroscopy was applied to identify a range of substances, including the detection of cement raw material content [16] and zearalenone and deoxynivalenol in maize [17], prediction of the *Ca* concentration in apples [18], content of 20 minerals in beef [19], residue amount of cyhalothrin in Brassica rapa [20], authentication and traceability of lemon [21], and so forth [22,23]. The success of these applications is based on the fact that NIR spectroscopy can simultaneously report a large number of analytes using the stretching vibrations of hydrogen groups (*X*-*H*, *X* is: *C*, *O*, *N*, *S*, etc.) and chemical bonds. Furthermore, this technique can be used to qualitatively identify the attribution of unknown samples even if there is only a subtle nuance in the composition of the samples themselves.

The orthogonal linear discriminant analysis (OLDA) algorithm [24,25] was rarely reported for dimension reduction of NIR data of pork meat samples. Additionally, three fuzzy clustering algorithms, namely fuzzy C-means (FCM) clustering [26], K-harmonic means (KHM) clustering [27,28], and fuzzy clustering with fuzzy covariance matrix (GK) [29,30,31], served as a classifier for the classification of spectra. For the fuzzy clustering algorithm, there is no need to provide the learning process of the training sample set, and it provides a new idea for the classification of pork meat storage times.

The objective of this research was to explore the combination of NIR diffuse reflectance spectroscopy and fuzzy clustering algorithms for the classification of pork meat based on the storage time. It is more fundamental to confirm that NIR coupled with fuzzy clustering algorithms can be used to distinguish the pork meat storage time before further exploring the feature extraction of NIR spectra by OLDA. Consequently, the detailed steps are as follows: (1) Use a NIR spectrometer to obtain the NIR spectrum of each pork meat sample at different storage times; (2) Pre-process the NIR spectra by multiplicative scatter correction (MSC) and reduce the dimensionality of the spectral data by OLDA; (3) Establish the classification models with FCM, KHM, and GK, and test their performance in classifying the pork meat samples at different storage times.

## 2. Materials and Methods

### 2.1. Sample Preparation

In this experiment, 67 pieces of pork meat samples were taken from the pigs weighing about 300 kg with the identical breed on the same farm and passed the standard slaughter (GB/T 17236-2019) and inspected by the health and quarantine department. All fresh pork meat samples labeled by serial number were selected from pig latissimus dorsi muscles (approximately 10 cm in length, 100 g each). The pork meat samples were packed with polyvinyl chloride (PVC) and transported to the laboratory at the ambient temperature of 18–25 °C. Considering that the pH value has a great impact on pork meat spoilage and microbial reproduction, we also measured the pH value every 12 h for each experiment to ensure that the difference between all samples was in the acceptable range of values. The pork meat samples were stored in a refrigerator at 0~4 °C. The NIR data of the samples were collected and recorded every 24 h during the 6 days [25,32]. As for reducing the error, each pork meat sample was sampled three times, and its average value served as the final testing data.

### 2.2. Spectral Data Acquisition

NIR spectroscopy technology has great potential in the field of food safety, so it has been rapidly developed. In this study, the Fourier transform near-infrared (FT-NIR) spectrometer Antaris II (Thermo Fisher Scientific Technologies, Co. Ltd., St. Louis, MO, USA) was used with embedded spectroscopic acquisition (sealed interferometer and room temperature deuterated triglyceride sulfate (DTGS) detector). In the process of spectra sampling, the spectral analysis system was sensitive to the temperature and moisture, so the temperature was maintained at 25 ± 1 °C and the relative humidity of air was also maintained at a relatively constant value (about 60%) in the sampling process. The wavenumber range of the spectra was 10,000~4000 cm^−1^ with a resolution of 4 cm^−1^. The light source was a high-stability halogen NIR light source. Each sample was scanned 32 times, each collection was repeated 3 times, and its average value was taken as the final detection data to obtain the mean diffuse reflectance spectrum, which is a 1557-dimensional datum.

### 2.3. Spectral Processing Method

Through spectral analysis of the above meat samples, the freshness information was analyzed. However, the baseline may drift due to physicochemical factors such as the dosage, reflection, temperature, concentration changes, and instrument anomalies. This is unsatisfactory as it does not meet the requirements of 100% transmittance, that is, an absorbance of zero, which has a negative impact on the calculation accuracy, spectral intensity, and classification results. In order to eliminate the influence of these factors and improve the signal-to-noise ratio, we chose a data preprocessing method commonly used in multi-wavelength calibration modeling, multiplicative scatter correction (MSC), thus enhancing the correlation between the spectrum and data. In practice, the average value of all spectral data is often assumed as the “ideal spectrum (standard spectrum)” [33] because the real ideal spectral data cannot be obtained:

(1) $$\vec{Data}=\frac{\sum_{i=1}^{n}Data_{i}}{n}$$

where *i* = 1, 2, …, *n*; and *n* is the number of samples.

### 2.4. Orthogonal Linear Discriminant Analysis

Linear discriminant analysis (LDA) is a classical feature extraction algorithm, and it is widely used in spectral data analysis. Nevertheless, LDA often faces the problem of a small sample size problem (SSSP) in practical applications [24], and SSSP refers to the singularity of the covariance matrix in the sample class. Orthogonal linear discriminant analysis (OLDA) uses a generalized linear discriminant algorithm framework, which redefines Fisher’s evaluation function by introducing the pseudo-inverse of the total scatter matrix *S_t_* [24]. OLDA eliminates noise interference between samples by orthogonalizing the feature vectors. OLDA is a dimension reduction technique of supervised learning, that is, each sample of its data set has a category output, which is different from PCA. The classical LDA technique in the field of pattern recognition (such as face recognition, ship identification, and image recognition) has a very wide application and can realize the data in low-dimension projection and make each of these categories as close as possible to the projection of points, and the category of the center distances between the different categories of data as big as possible.

Suppose k is the number of classes, G is a linear transformation, $S_{b}$ is the between-class matrix, $S_{w}^{}$ is the within-class scatter matrix, and $S_{t}^{}$ is the total scatter matrix.

Assume that it has a linear transformation G, so that [24]:

(2) $$S_{w}^{L}=G^{T}S_{w}G\;\;\;\;\;\;\;\;S_{b}^{L}=G^{T}S_{b}G\;\;\;\;\;\;\;\;S_{t}^{L}=G^{T}S_{t}G$$

By introducing a new discriminant function and using the generalized inverse to solve the singular problem, OLDA redefines Fisher’s evaluation function as [24]:

(3) $$F_{1}\left(G\right)=arg_{G}maxtrace\left({\left(S_{t}^{L}\right)}^{+}S_{b}^{L}\right)$$

After the calculation, a set of orthogonal projection vectors can be obtained, and they can meet the requirements of the OLDA algorithm for optimal projection vectors.

### 2.5. Fuzzy Clustering Algorithms

In this study, three fuzzy clustering algorithms (FCM, KHM, and GK) were used to characterize the component information in the spectral data. Fuzzy clustering divides a data set into different classes or clusters according to a specific standard (such as the distance criterion) to maximize the similarity of data objects in the same cluster and the difference with data objects not in the same cluster. Specifically, after clustering, data in the same category should be gathered as much as possible, and different kinds of data should be separated as much as possible.

#### 2.5.1. Fuzzy C-Means Clustering

FCM is the most widely used and successful fuzzy clustering algorithm. It computes the membership degree of each sample to all class centers by optimizing the objective function, and then determines the class of the sample to achieve the purpose of automatic classification of the sample data. In most cases, objects in a dataset cannot be divided into distinct clusters and assigning an object to a particular cluster is somewhat blunt and error prone. It is difficult to determine a suitable statistical model, so the use of the FCM with natural and improbability characteristics is an excellent choice. FCM is a clustering algorithm based on the minimum square error of the following equation:

(4) $$J_{FCM}=\sum_{k=1}^{N}\sum_{i=1}^{C}u_{ik}^{m}D_{ik}$$

where *N* is the number of samples, *C* is the number of data categories, *m* is the fuzzy weighting parameter, $u_{ik}$ is the fuzzy membership, and $D_{ik}$ is the Euclidean distance measure.

Based on the possibility constraint condition, the sum of the membership degree of the data points in all classes is 1.

#### 2.5.2. K-Harmonic Means Clustering

K-harmonic means clustering (KHM) is an iterative clustering method based on the objective function, taking the sum of the harmonic means of all data points to each cluster center as the objective function of clustering. Experiments have shown that KHM clustering is insensitive to the initial cluster centers due to the boosting function [27].

The fuzzy membership degree of KHM clustering [27] is:

(5) $$m\left(c_{j}|x_{i}\right)=\frac{{\left\|x_{i}-c_{j}\right\|}^{-p-2}}{\sum_{l=1}^{k}{\left\|x_{i}-c_{l}\right\|}^{-p-2}}$$

where $x_{i}$ is the i-th sample data, $c_{j}$ is the center of class j, p is the weight index, and k is the number of categories.

#### 2.5.3. Gustafson–Kessel Clustering

Gustafson–Kessel clustering can automatically cluster the data with different geometric shapes by calculating the fuzzy covariance matrix and the adaptive distance norm. The fuzzy membership degree and class centers of GK clustering are obtained by iterative calculation.

Then, the fuzzy membership degree $u_{ik}$ of sample $X_{k}$ belonging to class i is:

(6) $$u_{ik}={\left[\overset{c}{\underset{j=1}{\sum }}{\left(\frac{D_{ikAi}}{D_{jkAj}}\right)}^{\frac{2}{m-1}}\right]}^{-1},\forall i,k.$$

where $D_{ikAi}$ is the distance norm from sample $X_{k}$ to class center $V_{i}$, c is the number of categories, and m is the weight index, m∈(1,+∞).

### 2.6. Software

In this paper, the experimental software was designed and compiled by MATLAB 2018 (The MathWorks, Co. Ltd., Portolla Valley, CA, USA).

## 3. Results

### 3.1. NIR Spectral Analysis

Considering that the spoilage of pork meat is a complex process involving the growth of microorganisms, the spectra of pork meat samples under different storage time are also very complicated. We chose the random sampling method for data set division, and divided the sample set into two mutually exclusive parts to ensure there was consistency in the data distribution of the training set and testing set. In the above experiments, each pork meat sample was sampled every day over the course of six days. Then, 67 spectral data were collected every day as the sample data of one category; so, a total of 402 sample data from 6 categories were obtained. The six categories of sample data were divided into a training set and testing set, which was randomly selected according to a 2 to 1 ratio. The number of training set samples was 270, that is, 45 samples per day. The sample size of the testing set was 132, that is, 22 samples per day.

**Figure 1:**
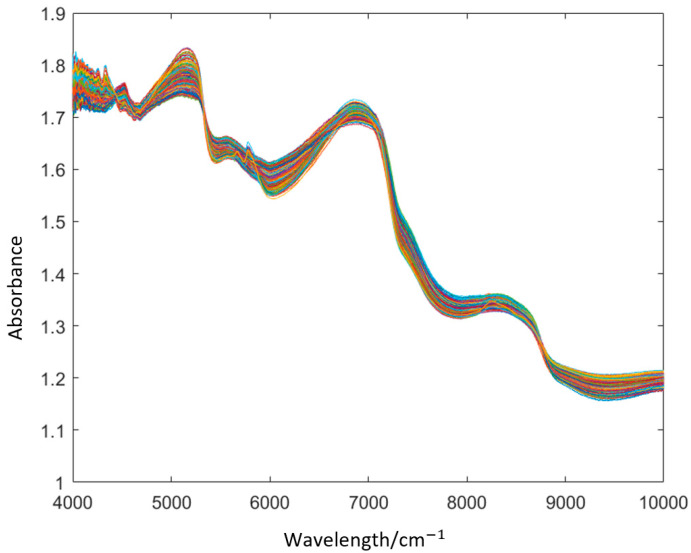
NIR spectra of the pork meat samples preprocessed by MSC.

The original spectral data of each sample were preprocessed using the MSC algorithm, and the baseline shift and offset of each spectrum were corrected with reference to the standard spectrum (the average of all spectral data). However, in the absorption spectrum, absorbance is also closely related to the sample particle size, uniformity, and tightness of the sample loading. The difference in the absorbance between different samples caused by granularity can be regarded as interference information, which is eliminated as much as possible by pretreatment during spectral analysis. Only a very small part of the spectra is related to differences in the composition (the organic molecules that constitute pork meat, such as protein, fat, and so on), so it can be regarded as the corresponding spectral absorption information, which has no influence on the whole data processing process, thus improving the spectral signal-to-noise ratio (SNR) [34]. Figure 1 demonstrates the NIR spectra of the pork meat samples preprocessed by MSC.

Due to the different storage times of the pork meat, there were differences in the NIR diffuse reflectance spectra, which helped in the correct identification of the pork meat storage time. It can be seen from Figure 1 that there were two peaks at about 5200 and 6900 cm^−1^, and spectral overlap occurred at the bottom of the peaks. Meanwhile, the spectral dispersion of the two bands of 4000~5100 and 5300~6800 cm^−1^ was satisfactory, indicating that these two bands mainly reflect the difference in the NIR diffuse reflectance spectra of the pork meat samples at different storage times. However, even these two bands with good dispersion still had some overlapped spectra, which caused difficulties in the classification of the NIR spectra, and some redundant information needed to be removed with the help of pattern recognition algorithms. The NIR spectra indicate the frequency doubling and combination information of molecular chemical bond vibration. The spectral data mainly reflect the information of the hydrogen-containing functional groups, such as C-H, N-H, and O-H, and the number of vibration absorption is directly related to it. The inorganic molecule in the spectral range of 4350–4000 cm^−1^ is H_2_O. Noise was produced in the sampling process, and a small area (4350–4000 cm^−1^) of spectral information after MSC pretreatment still contained noisy data, illustrating that the pork meat samples contained water. Since the purchased pork meat was fresh, and the water content was within the qualified range of the national standard (GB/T 17236-2019), the impact on the subsequent freshness test in the elaborations was minimal. In addition, the data dimensionality of these NIR diffuse reflectance spectra was up to 1557 dimensions, so OLDA was used to compress the dimensionality of the data and remove redundant information.

### 3.2. Orthogonal Linear Discriminant Analysis (OLDA) Model

**Figure 2:**
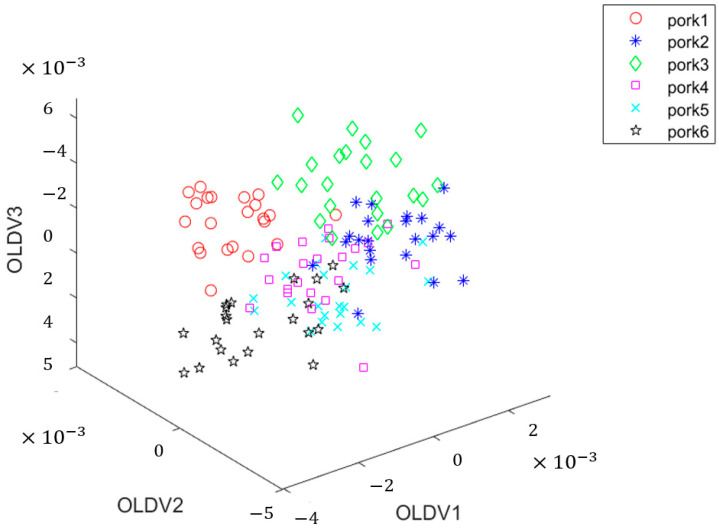
The score diagram of the three optimal discriminant vectors of OLDA.

OLDA can discriminate the orthogonal relations between projection vectors to eliminate redundant information in the projection, thus achieving stronger discriminant projection vectors than traditional discriminant projection vectors in terms of the recognition rate. OLDA was used to process the training set, and 1557-dimensional NIR diffuse reflectance spectra were compressed into 5-dimensional data. Three optimal discriminant vectors (OLDA1, OLDA2, and OLDA3) were obtained by selecting the first three-dimensional data computed by OLDA. The 5-dimensional data of 127 test samples were projected onto the above three vectors. Figure 2 shows the score diagram of the three optimal discriminant vectors.

Obviously, the data between the first day and the sixth day were separated, and the identification accuracy rate was high. However, regarding the data from the second day to the fifth day, there was some overlapped data, which were not easy to distinguish. It can be seen that the test sample distribution of the NIR spectral data was general, so the clustering accuracy was not satisfactory.

In order to better analyze the covariance matrix, the eigenvectors of the matrix were normalized and arranged in rows. Then, the eigenvalues were arranged from large to small, and the eigenvectors were arranged according to the eigenvalues. The eigenvectors corresponding to the loadings were the projection directions of the training set. Moreover, the NIR spectra had 1557 dimensions. After OLDA dimensionality reduction, they were compressed into five dimensions with five eigenvectors. In this study, the compressed NIR spectral data contained almost all the information of the original spectra and reduced the amount of computation for subsequent calculation. Meanwhile, the first component (OLDV1), the second component (OLDV2), and the third component (OLDV3) in Figure 2 explained 20% of the total variance, respectively. These three canonical scores accounted for 60% of the cumulative contribution rate in the training data.

### 3.3. Calculation of Fuzzy Membership

All parameters were set as follows: fuzzy weight index *m* = 2, maximum number of iterations *r_max_* = 100, termination threshold *t_h_* = 0.00001, and number of sample categories *c* = 6.

The initial cluster centers of FCM were:

(7) $$V^{\left(0\right)}=\left[\begin{array}{c} v_{1}^{\left(0\right)}\\ v_{2}^{\left(0\right)}\\ v_{3}^{\left(0\right)}\\ v_{4}^{\left(0\right)}\\ v_{5}^{\left(0\right)}\\ v_{6}^{\left(0\right)} \end{array}\right]=\left[\begin{array}{ccccc} -0.00052 & -0.00254 & 0.00187 & 0.00505 & -0.00191\\ -0.00182 & -0.00171 & 0.00064 & 0.00185 & -0.00310\\ -0.00112 & -0.00375 & 0.00145 & 0.00432 & -0.00253\\ -0.00131 & -0.00164 & 0.00008 & 0.00409 & -0.00134\\ -0.00198 & -0.00116 & 0.00116 & 0.00399 & -0.00373\\ -0.00217 & -0.00313 & 0.00131 & 0.00361 & -0.00294 \end{array}\right]$$

**Figure 3:**
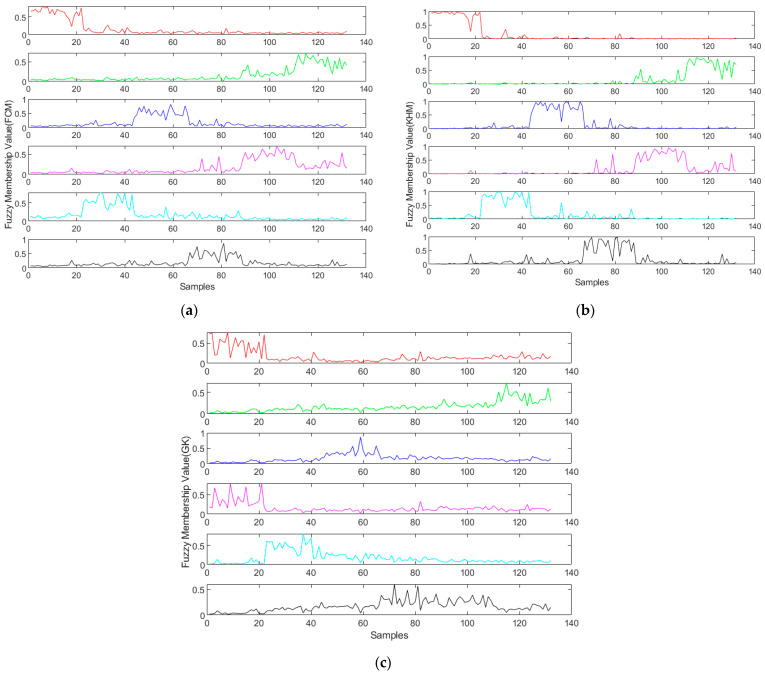
The fuzzy membership values of the samples in 6 days by fuzzy clustering algorithms: (**a**) FCM, (**b**) KHM, and (**c**) GK.

At first, FCM was performed to the convergence, and the fuzzy membership values and the cluster centers were computed. Then, GK and KHM were run with the cluster centers from FCM as their initial cluster centers. The fuzzy membership values of FCM, GK, and KHM are displayed in Figure 3. The abscissa is the *i*-th sample, and the ordinate represents the fuzzy membership value. In this experiment, there were 6 days of samples, representing day 1 to day 6 from top to bottom, so there were 6 subgraphs. If the fuzzy membership value of the *k*-th sample was the largest one in the *i*-th class, the *k*-th sample was taken as the sample belonging to the *i*-th class.

### 3.4. Results of the Fuzzy Clustering Algorithms

In this study, FCM, KHM, and GK clustering were used to classify pork meat samples at different storage times, and the clustering results of the different algorithms were compared. FCM, KHM, and GK are all iterative fuzzy clustering algorithms. They were initialized before running: The initial class centers of FCM clustering were the first six NIR data after OLDA dimension reduction. The number of samples was 127. The weight coefficient m = 2, p = 2, the number of categories c = 6, and the maximum number of iterations 100. The iteration termination criterion was 0.00001.

Table 1 shows the clustering results without pre-processing. As can be seen from Table 1, the clustering recognition rates obtained by FCM and GK were both 93.94%; however, the recognition rate of GK was only 87.88%. The number of iterations by GK was 45, which is bigger than those by FCM and KHM, so GK consumed more time than FCM and KHM. The results demonstrate that OLDA combined with FCM and KHM is a good strategy for the classification of pork meat storage times.

Table 2 shows the clustering results of FCM, KHM, and GK with the MSC preprocessing method. According to Table 2, the results are similar to those in Table 1; however, there are some changes in the recognition accuracy. The clustering recognition rates obtained by FCM and GK were both 93.18%; however, the recognition rate of GK decreased greatly, and it was only 65.90%. The number of iterations by GK reached 100 times, the maximum number of iterations we set to terminate. From the discriminant effect, KHM and FCM were more superior than the GK models. In terms of the number of iterations, KHM had the minimum number compared with the FCM and GK models. Combined with the graph of the fuzzy membership values, we believe that OLDA + KHM showed an excellent performance in the classification of pork meat storage times.

## 4. Discussion

In this study, the identification of pork meat storage times was investigated using FCM, KHM, and GK. We used NIR spectroscopy to achieve nondestructive detection of pork meat samples. Table 1 and Table 2 show the recognition results of the three fuzzy clustering methods. Overall, FCM and KHM were superior to GK, and the KHM model was the simplest in terms of the iteration times.

As for the high-dimensional NIR spectra, the OLDA algorithm was used to capture and reduce the discriminant information of the NIR spectra to obtain such an excellent discrimination effect. We can discuss it in detail from the following three aspects. First of all, as an effective method of analysis, NIR spectroscopy has high potential for the nondestructive detection of meat with a more stable power supply, signal amplifier, and more sensitive photon detector. Secondly, if pork products are affected by environmental factors for some hours, they will spoil. Then, the nutrients in pork meat (protein, etc.) gradually decompose, which is not conducive to human consumption. The resulting molecular material can be associated with specific absorption bands in the spectrum. Therefore, differences between samples (different storage times) can be reasonably distinguished. Finally, while some traditional methods may be more accurate and sensitive, they need supporting technicians, project funding, and high-precision experimental instruments. In addition, eliminating the use of complex chemical reagents can effectively avoid environmental pollution, greatly reducing the technical threshold of inspectors. Therefore, it is of great significance to develop a classification system for identifying pork meat storage times correctly and rapid use of the OLDA dimension reduction method combined with three fuzzy clustering algorithms.

This method can even be used on the assembly lines of large pork product factories [35,36,37]. Such an automated assembly line can ensure the authenticity, freshness, and safety of pork meat for each product. Furthermore, it can also be used for sampling detection by the Quality and Technology Supervision Bureau to identify whether the phenomenon of shoddy goods has occurred to avoid the behavior of some cheating consumers. Large meat-processing plants can calculate and classify large amounts of data using a computer [38], forming a professional sample database, which will greatly reduce the detection error rate. This major testing measure will also provide an incentive for these factories and enterprises to better comply with the country’s food safety standards. However, more experimentation and data collection work are needed before the technology can be correctly identified as a candidate for pork meat quality certification for large-scale use [39].

## 5. Conclusions

In order to discriminate pork meat storage times accurately and efficiently, we established a classification system for the identification of pork meat samples at different storage times based on NIR spectroscopy combined with OLDA and three fuzzy clustering algorithms. Firstly, 67 pork meat samples at different storage times were collected using an FT-NIR spectrometer. Secondly, the MSC method was applied to preprocess the NIR spectra to reduce noise and interference. Thirdly, OLDA, a dimension reduction method, was used to extract the discriminant information from the NIR spectra. Finally, under certain parameter settings, three fuzzy clustering algorithms (FCM, KHM, and GK) were performed to classify the meat samples. Both FCM and KHM indicated the highest classification accuracy of 93.18%, and in consideration of the iteration number, the KHM algorithm performed the best.

To sum up, the overall experimental results demonstrated that the combination of NIR spectroscopy and KHM has high potential in the discrimination of pork meat storage times. Since NIR spectroscopy has been widely used in the field of food science research, this scheme also provides feasibility for the identification of the freshness of other meat qualities.

## Figures and Tables

**Table 1:** The clustering results of FCM, KHM, and GK without pre-processing.

Methods	Number of Iterations	Misclassification Number	Accuracy
FCM	21	8	93.94%
KHM	1	8	93.94%
GK	45	16	87.88%

**Table 2:** The clustering results of FCM, KHM, and GK with the MSC preprocessing method.

Methods	Number of Iterations	Misclassification Number	Accuracy
FCM	32	9	93.18%
KHM	1	9	93.18%
GK	100	45	65.90%

## Data Availability

The data presented in this study are available on request from the corresponding author.
